# Newborn screening facilitates early theranostics and improved spinal muscular atrophy outcome: five-year real-world evidence from Taiwan

**DOI:** 10.1186/s13023-025-03697-1

**Published:** 2025-04-24

**Authors:** Chen-Hua Wang, Ting-Rong Hsu, Mei-Ying Liu, Li-Yun Wang, I-Jun Chou, Wang-Tso Lee, Wen-Chen Liang, Inn-Chi Lee, Hsiao-Jan Chen, Shu-Min Kao, Hui-Chen Ho, Dau-Ming Niu, Kwang-Jen Hsiao, Ming-Yuh Chang, Hui-Min Hsieh, Yuh-Jyh Jong

**Affiliations:** 1 Department of Pediatrics, Kaohsiung Municipal Siaogang Hospital, Kaohsiung Medical University, Kaohsiung, Taiwan; 2 Department of Pediatrics, Kaohsiung Medical University Hospital, Kaohsiung Medical University, Kaohsiung, Taiwan; 3 Translational Research Center of Neuromuscular Diseases, Kaohsiung Medical University Hospital, Kaohsiung Medical University, Kaohsiung, Taiwan; 4 Department of Pediatrics, Taipei Veterans General Hospital, Taipei, Taiwan; 5 School of Medicine, College of Medicine, National Yang Ming Chiao Tung University, Taipei, Taiwan; 6 Neonatal Screening Center, The Chinese Foundation of Health, Taipei, Taiwan; 7 Neonatal Screening Center, Taipei Institute of Pathology, Taipei, Taiwan; 8 Department of Pediatric Neurology, Chang Gung Memorial Hospital, Linkou Branch, Taoyuan, Taiwan; 9 Department of Pediatric Neurology, National Taiwan University Children’s Hospital, Taipei, Taiwan; 10 Department of Pediatrics, and Graduate Institute of Brain and Mind Sciences, College of Medicine, National Taiwan University, Taipei, Taiwan; 11 School of Medicine, College of Medicine, Kaohsiung Medical University, Kaohsiung, Taiwan; 12 Graduate Institute of Clinical Medicine, College of Medicine, Kaohsiung Medical University, Kaohsiung, Taiwan; 13 Division of Pediatric Neurology, Department of Pediatrics, Chung Shan Medical University Hospital, Taichung, Taiwan; 14 Institute of Medicine, School of Medicine, Chung Shan Medical University, Taichung, Taiwan; 15 Institute of Clinical Medicine, College of Medicine, National Yang Ming Chiao Tung University, Taipei, Taiwan; 16 Department of Medical Research, Taipei Veterans General Hospital, Taipei, Taiwan; 17 Preventive Medicine Foundation, Taipei, Taiwan; 18 Department of Education and Research, Taipei City Hospital, Taipei, Taiwan; 19 Pediatric Neurology, Changhua Christian Children’s Hospital, Changhua, Taiwan; 20 Department of Public Health, Kaohsiung Medical University, Kaohsiung, Taiwan; 21 Department of Laboratory Medicine, Kaohsiung Medical University Hospital, Kaohsiung Medical University, Kaohsiung, Taiwan; 22 Center for Neurotechnology, Kaohsiung Medical University, Kaohsiung, Taiwan; 23 Department of Biological Science and Technology, National Yang Ming Chiao Tung University, Hsinchu, Taiwan

**Keywords:** Spinal muscular atrophy, Newborn screening, Early treatment, Symptomatic treatment, Presymptomatic treatment, Disease-modifying therapy, Survival motor neuron 1 gene, Survival motor neuron 2 gene copies, Clinical outcome, Real-world evidence

## Abstract

**Background:**

Recent findings indicate that infants with spinal muscular atrophy (SMA) treated early through newborn screening (NBS) have better outcomes. This study aimed to investigate the long-term outcomes of a 5-year SMA NBS program in Taiwan.

**Results:**

From September 2017 to August 2022, two NBS centers screened patients for *SMN1* homozygous deletion using quantitative real-time polymerase chain reaction (RT-PCR) or the Sequenom MassARRAY platform and subsequently confirmed the findings using multiplex ligation-dependent probe amplification (MLPA). Implementation of SMA NBS using RT-PCR or MassARRAY platform efficiently led to the detection of neonates with homozygous survival motor neuron 1 (*SMN1*) deletions at a median age of 9 (range 4–14) days. Among the 446,966 newborns screened, 22 were detected to have a homozygous deletion of *SMN1,* followed by MLPA confirmation. One patient initially showed negative screening results but was later confirmed to have a compound heterozygous mutation. Among the 23 confirmed cases, 8 patients had two *SMN2* copies (all classified as SMA type 1), 11 patients had three *SMN2* copies (including 4 with SMA type 1, 2 with SMA type 2, 3 with SMA type 3, and 2 asymptomatic cases), and 4 patients had four *SMN2* copies (all asymptomatic). The mean (median) follow-up duration for 19 survivors was 4.2 (5.0) years. All patients with two *SMN2* copies developed symptoms within 62 days; those with three *SMN2* copies experienced disease onset within 1 year. After diagnosis, most patients were on a watch and wait to receive disease-modifying therapy (DMT) due to initial lack of insurance coverage and limitations on indications after coverage was granted. Of the 19 children who received DMT, the outcomes included 12 walkers, 1 walker requiring support, 3 sitters, 1 non-sitter, and 2 patients with SMA type 1b with two *SMN2* copies who succumbed to acute respiratory failure.

**Conclusions:**

This 5-year SMA NBS study using RT-PCR or the MassARRAY platform, along with an extended follow-up, demonstrates that early diagnosis and prompt treatment can enhance SMA clinical outcomes and change its natural progression in the therapeutic era. Infants with NBS who received presymptomatic DMT had better clinical outcomes than those who received symptomatic DMT.

**Supplementary Information:**

The online version contains supplementary material available at 10.1186/s13023-025-03697-1.

## Background

Spinal muscular atrophy (SMA) is an autosomal recessive neuromuscular disease characterized by survival motor neuron 1 (*SMN1*) gene mutation and degeneration of spinal motor neurons, resulting in generalized muscle hypotonia, weakness, and atrophy [1]. SMA is the leading cause of infant mortality due to genetic disease worldwide [2]. Based on the age at onset and best-achieved motor function, SMA is classified into four clinical subtypes (types 1, 2, 3, and 4) [3]. SMA type 1 (SMA1, MIM# 253,300) is the most severe form; patients become symptomatic before 6 months of age and rarely survives beyond 2 years of age. SMA1 can be further classified into 1a, 1b, and 1c according to the age at onset (birth, < 3 months, and 3–6 months, respectively) [4]. SMA type 2 (SMA2, MIM# 253,550) has an intermediate severity, characterized by onset before 18 months of age and an inability to walk unaided. SMA2 can be further classified into 2a or 2b according to the age of onset (after 6 months or 1 year) [4]. SMA type 3 (SMA3, MIM#253,400) is a mild form, characterized by a late childhood onset and the ability to walk unaided. SMA3 can be further classified into 3a or 3b according to the age of onset (before or after 3 years of age) [4]. SMA type 4 (SMA4; MIM#271,150) is the adult-onset form.

The worldwide incidence of SMA is estimated to be approximately one in 14,300 individuals [5]. The incidence of SMA in Taiwan is estimated to be 1 in 17,181 [6], and the carrier frequency is 1 in 48 [7]. Approximately 95% of SMA cases are caused by the homozygous loss of exon 7 via deletion or gene conversion [1]. The paralogous gene, survival motor neuron 2 (*SMN2*), also encodes the SMN protein and differs from *SMN1* by the presence of five nucleotides, including 835-44G>A, c.840C>T, c.888+100A>G, c.888+215A>G, and c.1155G>A [8]. However, the c.840C>T change in exon 7 of the *SMN2* results in the preferential exclusion of exon 7 during splicing, and only 5–10% of translated proteins are functional. Patients with SMA and high *SMN2* copy numbers generally have a milder phenotype [9, 10].

Since 2016, the United States Food and Drug Administration-approved three SMA disease-modifying therapies (DMTs) have been introduced, including antisense oligonucleotide (nusinersen), gene therapy (onasemnogene abeparvovec), and oral small molecule (risdiplam) [11]. The treatment outcomes of patients with SMA depends on *SMN2* copy numbers, disease duration, pre-treatment motor function, and age at treatment initiation [12]. Existing studies have demonstrated that presymptomatic infants at risk for SMA who received DMTs had better motor and non-motor outcomes than did symptomatic infants with SMA who received DMTs [13]. Therefore, newborn screening (NBS) is a vital program that allows physicians to promptly diagnose and treat SMA in the therapeutic era. At present, the treatment approach for SMA comprises immediate intervention for patients with two or three *SMN2* copies upon diagnosis [14]. For patients with four copies, the decision remains controversial; however, a recent expert consensus suggests prompt treatment initiation [15].

In Taiwan, nusinersen was initially used as a DMT via an expanded access program in 2017 and later became the first DMT approved by the National Health Insurance (NHI) in 2020. Risdiplam and onasemnogene abeparvovec were not reimbursed by the Taiwan NHI until April 2023 and August 2024, respectively; however, these two drugs were made available by participating in global clinical trials or compassionate use programs during the study period. Owing to the initial lack of insurance coverage and limitations on indications after coverage was granted, some patients in this cohort did not receive treatment immediately after diagnosis.

A worldwide survey published in 2021 identified only nine SMA NBS programs across 152 countries. Taiwan was recognized as the only country offering a nationwide SMA NBS program, along with access to DMT. By 2023, 31 countries had established such programs, covering 7% of newborns worldwide. In countries without an SMA NBS program or DMT coverage, most infants diagnosed with SMA are placed on a “watch and wait” approach, hoping to start DMT as soon as it becomes available [16]. However, there remains a lack of detailed real-world data on the long-term outcomes of NBS patients, particularly regarding SMA type and treatment modality. This study aimed to evaluate the outcome of a 5-year SMA NBS program utilizing quantitative real-time polymerase chain reaction (RT-PCR) and MassARRAY platforms. In addition, we sought to analyze the long-term outcomes of 22 patients with homozygous *SMN1* deletions and one patient with compound heterozygous pathogenic variants in *SMN1*. The analysis will include data on *SMN2* copy numbers, selected treatment modalities, and motor and non-motor outcomes in a real-world setting.

## Methods

### Participants

A nationwide SMA NBS study was first initiated by Kaohsiung Medical University Hospital (KMUH) in Kaohsiung, Taiwan, in collaboration with two of three NBS centers in Taiwan: the Chinese Foundation of Health (CFOH) and Taipei Institute of Pathology (TIP) (ClinicalTrials.gov Identifier: NCT03217578; KMUH IRB No.: KMUHIRB-SV(II)−20160036). The program, launched on September 1, 2017, was subsequently integrated into the NBS workflow at both the CFOH and TIP. Parents who provided informed consent were invited to participate in the program. All signed informed consent forms were securely stored in delivery clinics or hospitals, following the standard procedures of the respective NBS centers. Dried blood spot (DBS) samples were routinely collected from newborns at 48 h of life or 24 h after the first feeding using a heel prick to spot blood onto filter papers. The DBS samples labeled for SMA testing were screened for *SMN1* exon 7 homozygous deletion. This study protocol was registered at ClinicalTrials.gov on July 12, 2017, with the first infant enrolled on September 1, 2017. The screening program included approximately 64% of newborns in Taiwan, with a total of 446,966 infants screened between September 1, 2017 and August 31, 2022. Of these, 37 neonates tested positive for SMA during the initial screening, and their results were subsequently confirmed using multiplex ligation-dependent probe amplification (MLPA). Of the 37 SMA-positive neonates, 22 were identified to have homozygous *SMN1* deletions. In addition, one infant who initially tested negative during screening was later confirmed to have compound heterozygous mutations via *SMN1* sequencing.

To collate information on the treatments and clinical progression of the 23 cases with *SMN1* deletions or mutations, we conducted a prospective and retrospective comprehensive chart review at three medical centers: KMUH in Kaohsiung, Taiwan (IRB No. KMUHIRB-SV(II)−20200014 and KMUHIRB-E(I)−20240087); Chang Gung Memorial Hospital in Linkou, Taoyuan (CGMH), Taiwan (IRB No. 202202354B0); and Taipei Veterans General Hospital in Taipei (TVGH), Taiwan (IRB No. 2024-01-005A). We instructed parents and caregivers to contact us immediately if they observed any of the following: a significant change in the child’s movement, feeding, or breathing patterns during illness; a decline or loss of previously attained motor abilities or failure to achieve expected motor milestones; difficulty feeding or breathing, particularly in young children or infants; or increased fatigue unrelated to activity levels [14]. Doctors at the three medical centers followed up on positive cases identified through NBS according to the following schedule: weekly communications for the first 6 months, monthly from six to 12 months, bimonthly until 3 years of age, and semi-annually thereafter. In total, 23 positive cases were followed up across the three medical centers, with 17 cases at KMUH, 5 at TVGH, and 3 at CGMH.

Patients who received DMT at KMUH were invited to participate in a prospective follow-up study, with their parents providing informed consent. For patients receiving DMT at CGMH and TVGH, those who did not receive DMT, participated in global trials, or died, the medical records were collected retrospectively. The chart review was conducted from September 12, 2017 to July 31, 2023.

### Screening methods

Two genotyping platforms were used: the TaqMan quantitative RT-PCR assay (Supplemental Fig. 1) by the CFOH and the Sequenom MassARRAY assay (Supplemental Fig. 2) by TIP. For RT-PCR assays, primers and probes targeting *SMN1* c.888 + 100 A were used during the first year of screening. Subsequently, the target was changed to c.840 C. The sequences of the primers and probes used to target c.888 + 100 A and c.840 C have been previously published [6, 17]. Samples with *SMN1* crossing point (Cp) ≥ 30 or < 20 were retested for both *SMN1* and *ACTB* using DNA extracted from independent punches. Samples without *SMN1* and with an *ACTB* Cp < 29 were considered positive.

**Fig. 1:**
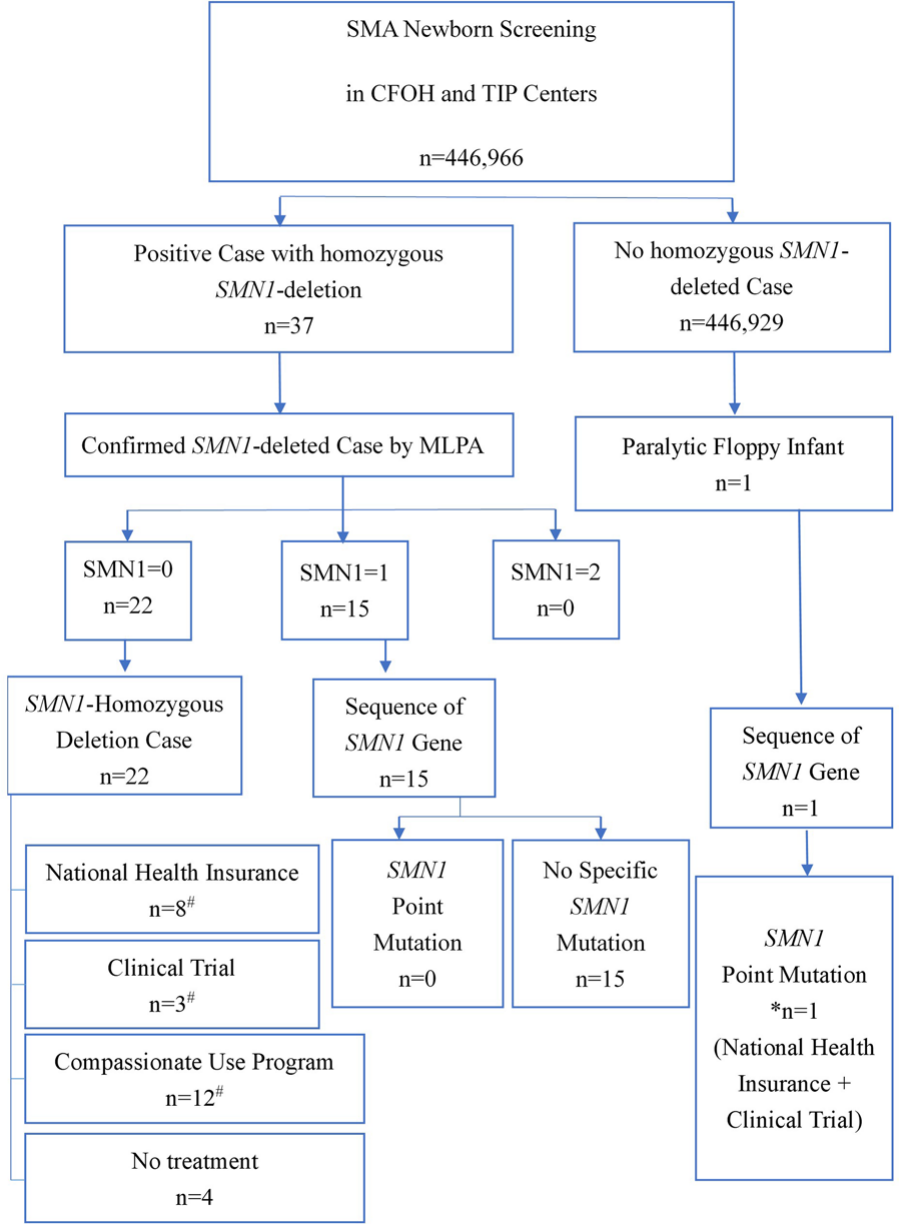
Flowchart of SMA newborn screening. SMA: spinal muscular atrophy; CFOH: Chinese Foundation of Health; TIP: Taipei Institute of Pathology; *SMN1*: Survival Motor Neuron 1; MLPA: Multiplex Ligation-Dependent Probe Amplification; *: *SMN1* sequence with deletion of one *SMN1* allele and c.22dupA frameshift mutation in another allele; ^#^: One patient received national health insurance followed by a clinical trial, and four patients received both national health insurance and the compassionate use program for disease- modifying therapy

**Fig. 2:**
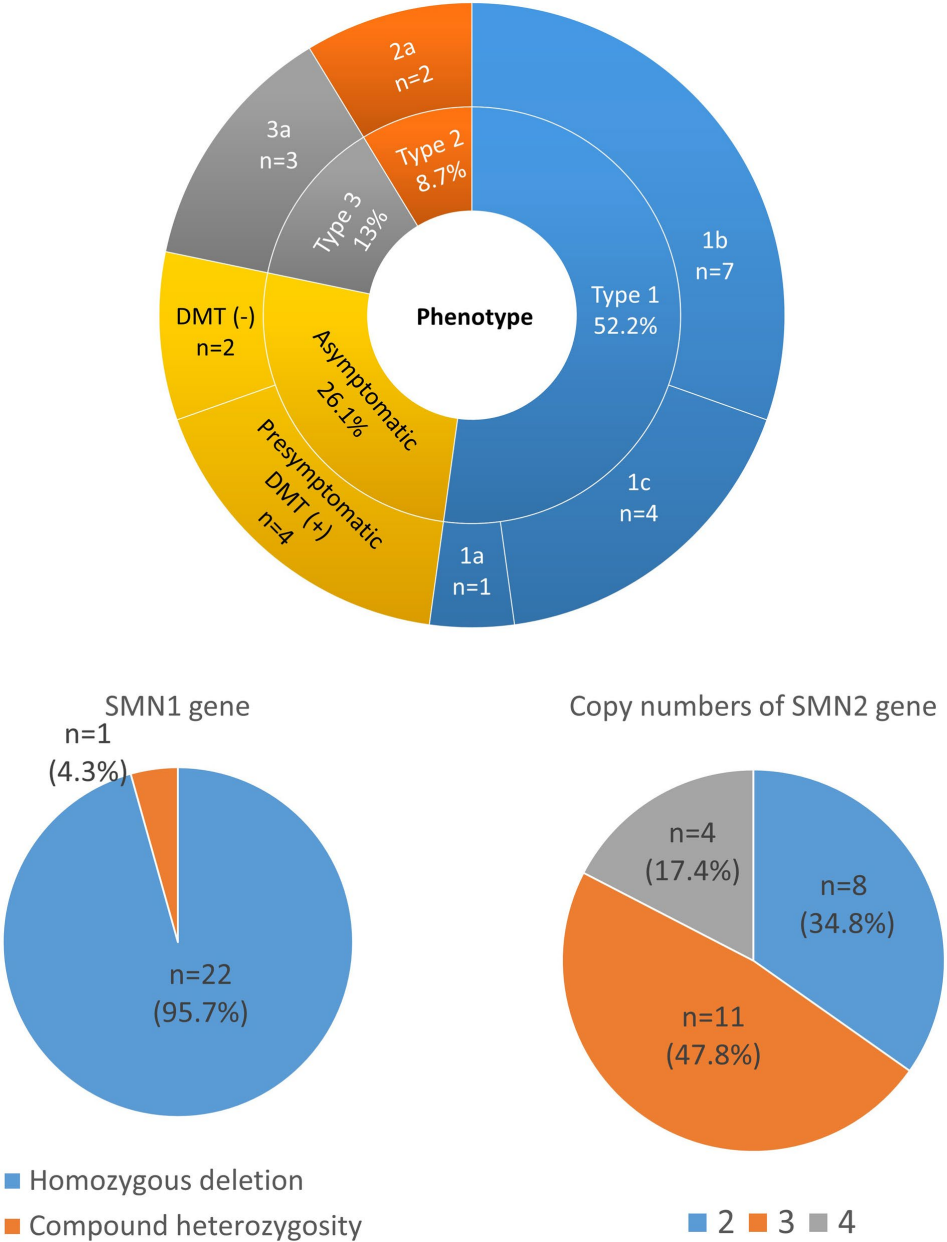
Phenotypes and genotypes of patients with SMA. SMA: spinal muscular atrophy

TIP used the MassARRAY assay with the SMA NeoScreening kit (Feng Chi Biotech Corp. Taipei, Taiwan) to detect homozygous *SMN1* deletion [18, 19]. In each multiplex PCR, amplicons targeting four *SMN1*/*SMN2* variants of interest, including the c.840 and c.1155 nucleotides, were amplified. Afterward, a MassARRAY mass spectrometer (MassARRAY Analyzer 4, Agena Bioscience, San Diego, CA, USA) was used to collect mass spectra from the time-resolved data. These spectra were analyzed using the SpectroTYPER software (Agena Bioscience, San Diego, CA, USA) to perform genotype calling. *SMN1* absence was determined based on SMA allele ratios, which were estimated from *SMN1* and *SMN2* signal intensities. A ratio ˃0.85 was considered positive for *SMN1* deletion.

### Confirmatory testing

The screening flowchart is shown in Fig. 1. Patients that showed positive screening results were referred to one of the four referral hospitals (TVGH, Changhua Christian Children Hospital, Chung Shan Medical University Hospital, and KMUH) for initial evaluation and confirmation. Peripheral blood samples were collected to determine the copy numbers of *SMN1* and *SMN2* using MLPA. A zero copy of *SMN1* exon 7 confirmed the diagnosis of SMA. After confirmatory diagnosis, all patients with zero copies of the *SMN1* gene were followed up by pediatric neurologists at CGMH, TVGH, and KMUH.

### Clinical data

We retrospectively reviewed the medical records of probands who were confirmed to be at risk of SMA via MLPA or *SMN1* gene sequencing. Age at SMA onset, *SMN1* deletion or mutation, *SMN2* copy numbers, clinical type, age at death, age at initial DMT, DMT use through three sources, including national health insurance, compassionate use program, and clinal trials, ventilator use, tube feeding, and motor outcomes were analyzed. Motor outcomes were classified into six categories: non-sitter, sitter, walker with support, walker, asymptomatic walker with or without receiving DMT, and death.

### Data availability statement

The authors certify that this manuscript reports clinical trial data (NCT03217578). Individual patient data will not be publicly released but a portion of the SMA NBS data was presented at the 66 th Annual Meeting of the Japanese Society of Child Neurology in Nagoya, Japan on May 30, 2024. The protocol and data supporting the findings of this study are available from the corresponding author upon reasonable request.

### Statistical analysis

Descriptive analyses were performed to present the frequency results of the SMA NBS program and the case characteristics between the two SMA NBS centers in Taiwan. The cumulative incidence rates among newborns were used to determine whether there was a statistically significant difference between the incidence rates at the two centers. Confidence intervals were calculated using Poisson distribution [20]. Regarding the comparison of screening methods, the sensitivity and specificity of each screening approach were calculated. The Wilson score interval approach, which is commonly used when dealing with small samples and skewed observations, was used to estimate the 95% confidence intervals (CIs) [21]. Fisher’s exact test was used to assess the difference in c.1-39A>G variations between the distribution of the normal and hybrid alleles, with a *P*-value < 0.01 considered significant.

## Results

### Demographic characteristics of screened newborns

From September 1, 2017 to August 31, 2022, 64.3% (536,703 out of 835,164) of live births in Taiwan were screened by the CFOH and TIP NBS centers. Of these, 83.3% (446,966 of 536,703) underwent SMA testing (Table 1).

**Table 1:** Screening results of two SMA newborn screening centers in Taiwan

NB screening centers	CFOH	TIP	Total
SMA Screening Methods	RT-PCR	Sequenom MassARRAY	
No. of Total NB#	271,333	265,370	536,703
No. of Screened NB	236,214 (87.1%)	210,752 (79.4%)	446,966 (83.3%)
No. of Referred Cases	13	24	37
Male	3	12	15
Female	10	12	22
Confirmatory Method	Multiplex Ligation-Dependent Probe Amplification (MLPA)		
No. of *SMN1* = 0	9	13	22
Male	2	6	8
Female	7	7	14
No. of *SMN1* = 1	4	11	15
Male	1	6	7
Female	3	5	8
No. Confirmed NB	9	14*	23
Incidence	1/26,246^@^	1/15,054^@^	1/19,433

^#^ Total live newborns in Taiwan during the 5-year screening period are 835,164; *One infant with a screening-negative result became symptomatic at 1 month of age, and results of MLPA and SMA gene sequencing indicated that this case was a compound heterozygote with one exon 7 deletion and c.22 dupA frameshift mutation on the other allele with two *SMN2* copies, which was beyond the scope of this detection. Screening period: from September 1, 2017 to August 31, 2022. ^@^There was no statistical difference between the incidences of SMA estimated by the two NBS centers

SMA, spinal muscular atrophy; NB, newborn; NBS, newborn screening; CFOH: Chinese Foundation of Health; TIP: Taipei Institute of Pathology

### Screening results

In both NBS centers, > 99.9% of the neonates had normal SMA screening results without the need for retesting. A total of 37 cases screened positive; both RT-PCR and the MassARRAY platform can efficiently detect neonates with homozygous *SMN1* deletion at a median 9 (range 4–14) days of life (DOL). Except for two infants who required resampling due to poor sample quality, the majority of infants (35/37, 94.6%) were transferred to the referred hospitals at a median DOL of 10 (range 5–14) days. All 37 screening-positive infants underwent confirmatory MLPA testing. Confirmation of diagnosis and determination of *SMN2* copy numbers were reported at a median DOL of 21 (range 10–63, delay in five infants: two were due to a retest and three by parental request) days. Four and eleven screening false-positive cases were detected by RT-PCR and MassARRAY spectrometry, respectively. Sequence examination of the 15 infants with one copy of *SMN1* revealed that 8 had *SMN1*-*SMN2* hybrid alleles (Supplemental Fig. 3) and 7 had sequence variations that interfered with detection.

**Fig. 3:**
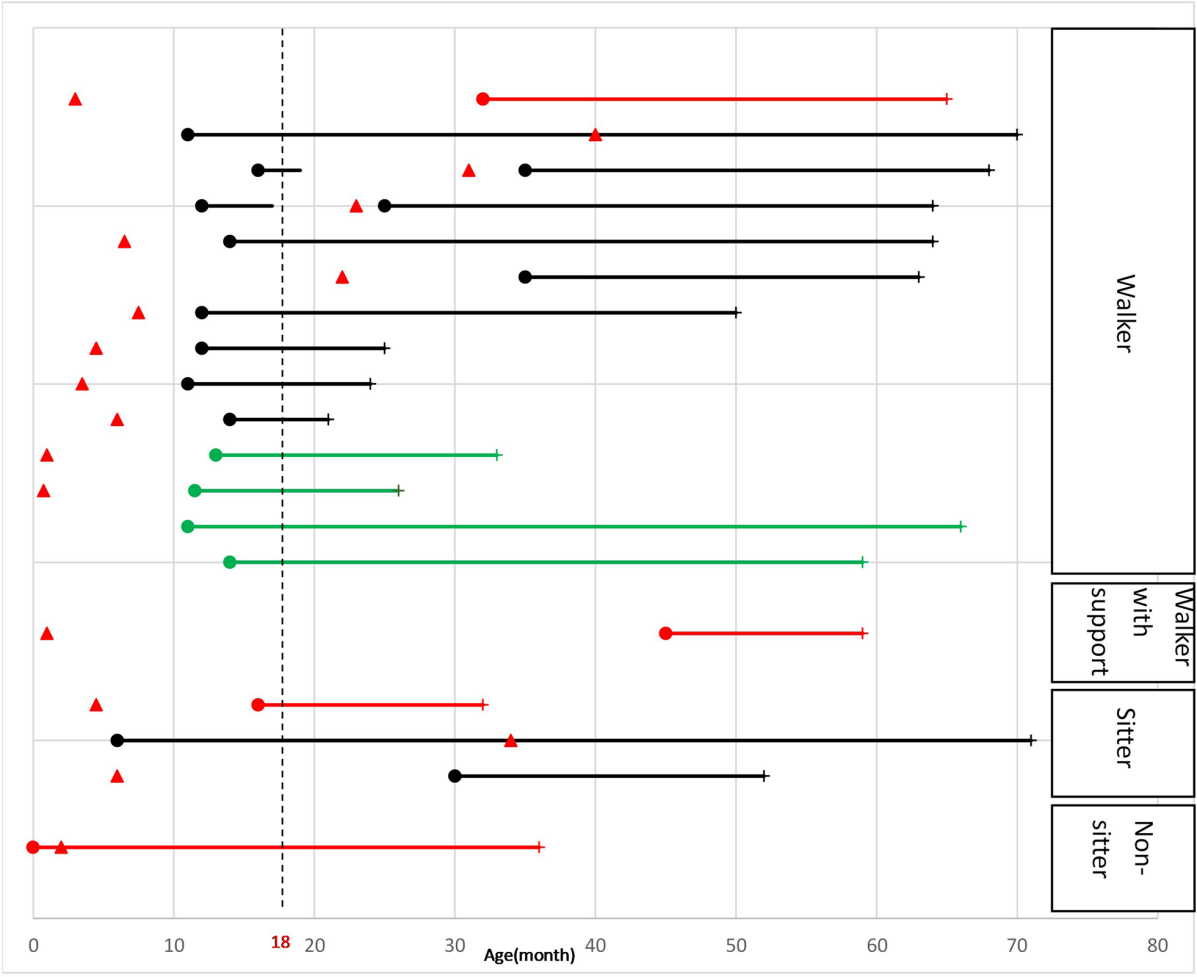
Motor development in our patients (death is excluded). Each line represents an individual patient. ●: Age at the highest milestone achievement; ▲: Age at initial DMT; + : Current age. The red line represents patients with two *SMN2* copies; the black line represents patients with three *SMN2* copies; and the green line represents patients with four *SMN2* copies. DMT: disease-modifying therapy

Notably, one case with a screening-negative result became symptomatic at 1 month of age. The results of MLPA and *SMN1* gene sequencing indicated a compound heterozygote with one exon 7 deletion and a c.22dupA frameshift mutation in the other allele, which could not be identified by the two NBS methods used in this study. The overall incidence of SMA in the present study was 1/19,433 (95% CI, 1/12,950 - 1/29,162).

### Clinical characteristics and motor and non-motor outcomes of SMA-confirmed cases

Prospective and retrospective follow-ups (from September 12, 2017 to July 31, 2023) of 23 SMA-confirmed cases (9 males and 14 females) showed that 8 had two *SMN2* copies (34.8%), 11 had three copies (47.8%), and four had four copies (17.4%). (Table 2) The mean (median) follow-up duration of this study was 4.2 (5.0) years, ranging from 1.8 to 5.9 years (excluding the four death cases).

**Table 2:** Demographics, phenotypes, genotypes, and motor and non-motor outcomes of patients with SMA

*SMN1*:*SMN2* copy number	0:2 (n = 8)	0:3 (n = 11)	0:4 (n = 4)
Sex, n (%)			
Male	4(50%)	3(27.3%)	2(50%)
Female	4(50%)	8(72.7%)	2(50%)
SMA type, n (%)			
Type 1a	1(12.5%)		
Type 1b	7(87.5%)		
Type 1c		4(36.3%)	
Type 2a		2(18.2%)	
Type 3a		3(27.3%)	
Asymptomatic		2(18.2%)	4(100%)
Age at first symptom onset, mean ± SD (range)	33.6 ± 20.2 (1–62) days	8.4 ± 3.0 (4.5–11.7) months	N/A
Age at initial DMT, mean ± SD (range), months	2.5 ± 1.1(1–4.5)	type 1: 6.5 ± 0.6 (6–7.5) type 2: 28 ± 6 (22–34) type 3: 31.3 ± 6.9 (23–40)	0.9 ± 0.1 (0.75–1)
DMT, n (%)			
Nusinersen	2(25%)	4(36.3%)	
Onasemnogene abeparvovec		4(36.3%)	
Risdiplam			2(50%)
Switch	4(50%)	2(18.2%)	
Add-on		1(9.1%)	
None	2(25%)		2(50%)
Motor outcome			
Non-sitter	1(12.5%)		
Sitter	1(12.5%)	2(18.2%)	
Stand unaided			
Walker with support	1(12.5%)		
Walker unaided	1(12.5%)	9(81.8%)	4(100%)
Death#	4(50%)		
Ventilator ever used	7(87.5%)	0(0%)	0(0%)
Tube feeding ever used	7(87.5%)	0(0%)	0(0%)

^#^Two patients received palliative care and two received disease-modifying therapy

*SMA*, Spinal muscular atrophy; *DMT*, Disease-modifying therapy; *SD*, Standard deviation

### Infants with two-copy *SMN2* (n = 8)

One patient with SMA1a was severely affected at birth, presenting with acute respiratory distress, dysphagia, paralytic floppiness, right frontoparietal and left parietal subdural hematomas, and an atrial septal defect. As per parental request, nusinersen was started at approximately 1–3 months of age, followed by risdiplam around 1–1.5 years of age. The child maintained a non-sitter status, relied on a 24 h ventilator through tracheostomy, and was fed totally by a nasogastric tube; in addition, the child had marked limb weakness with minimal finger movement, bilateral ventriculomegaly with a thin corpus, and recurrent disseminated skin ulcerated lesions with coagulative necrosis and vasculopathy. In addition, seven infants had SMA1b with two *SMN2* copies, including one initial screening-negative infant who was later confirmed to have a compound heterozygous mutation (c.22dupA frameshift mutation); all of these seven infants became symptomatic at a median DOL of 30 (range 18–62 days). Among them, two families chose palliative care and deceased within 1 year of age. The other five infants received DMT after the onset of clinical symptoms owing to a lack of health insurance coverage at the time. Three patients received switch therapy (nusinersen followed by onasemnogene abeparvovec), and the ability to walk independently developed in only one patient at 2 years and 8 months of age (Fig. 3). Furthermore, two nusinersen-treated patients died of acute respiratory distress at a median age of 12.8 months.

### Infants with three-copy *SMN2* (n = 11)

Among the 11 patients with three *SMN2* copies, two infants (2/11, 18.2%) received presymptomatic DMT at a median of 123 DOL, and both could walk independently before 18 months of age with normal motor milestones. The other nine patients (9/11, 81.8%) were treated after symptom onset; of these, seven were able to walk independently but only four achieved this ability before 18 months of age. Before DMT initiation, one patient with SMA 3a patients lost their walking ability at 1 year and 7 months of age and another lost the ability at 1 year and 9 months of age. After DMT, the patients regained independent ambulation at 2 years and 11 months and 2 years and 9 months of age, respectively (Fig. 3). The median onset ages of SMA1c, SMA2a, and SMA3a were 158 DOL (range 135–165 days), 322.5 DOL (range: 311–334 days), and 330 DOL (range 330–350 days), respectively.

### Infants with four-copy *SMN2* (n = 4)

Two infants with four *SMN2* copies received DMT and remained asymptomatic walkers at a median age of 2.6 years. In addition, two children with four *SMN2* copies, with a median age of 5.3 years, did not receive DMT and remained asymptomatic walkers (Fig. 3).

### Incidence of SMA

The average 5-year incidence of SMA was 1 in 19,433 live births. Notably, a significant decline in SMA incidence was observed over consecutive 5-year SMA NBS periods. From September 2017 to August 2018, the incidence was 1 in 8516, which decreased to 1 in 70,015 between September 2021 and August 2022 (Table 3).

**Table 3:** SMA incidence from NBS between September 2017 and August 2022

Period of SMA NBS	No. of NB		Screening rate (%)	Confirmed SMA cases	Incidence
	Total NB	SMA screened			
2017.9–2018.8	118,348	110,705	93.5	13	1/8,516
2018.9–2019.8	114,202	99,826	87.4	2	1/49,913
2019.9–2020.8	107,499	85,936	79.9	3*	1/28,645
2020.9–2021.8	103,193	80,484	78.0	4	1/20,121
2021.9–2022.8	93,461	70,015	74.9	1	1/70,015
2017.9–2022.8	536,703	446,966	83.3	23	1/19,433

*SMA*, Spinal muscular atrophy; *NBS*, Newborn screening. *One SMA NBS-screened negative case was clinically and genetically confirmed. The MLPA assay confirmed that the patient had only one *SMN1* copy. The *SMN1* gene sequencing revealed that the patient harbored a c.22dupA frameshift mutation

## Discussion

This study presents the result of an SMA NBS program for five consecutive years in Taiwan, which included 22 *SMN1* homozygous deletion cases and one case with *SMN1* compound heterozygosity from 446,966 newborns. The *SMN1* mutation rate was 4.3% (1/23), consistent with the fact that 5% of SMA cases are caused by point mutations [1]. A limitation of the SMA NBS is that infants with compound heterozygosity involving *SMN1* point mutations cannot be identified using RT-PCR and Sequenom MassARRAY platforms. Therefore, physicians should consider the possibility of SMA in hypotonic infants or children with motor delays and hyporeflexia or areflexia, even if the SMA NBS results are normal, and pursue differential diagnosis through MLPA or *SMN1* sequencing. NBS-positive data (Supplemental Table 1) obtained in the first year assisted NBS centers in optimizing NBS methods and refining interpretation methods (Supplemental Table 2). The possibility of discovering hybrid alleles and false-positive cases is minimized through the optimization of oligonucleotide and probe sequence design or by directly determining the genotype of c.840. This optimization has reduced clinically unnecessary burden and alleviated parental concerns. To accelerate the diagnostic process and provide essential information on *SMN2* copy numbers for treatment planning, we propose integrating MLPA testing with patient referrals at NBS centers. This approach will enable timely treatment and improve therapeutic outcomes for patients.

Our study demonstrates that SMA NBS performed using RT-PCR or MassARRAY is feasible for detecting presymptomatic neonates with homozygous *SMN1* deletion at risk for SMA shortly after birth, except in one neonate with SMA1a. Both methods demonstrated high-throughput genotyping capabilities, rapid experimental timelines, and applicability in clinical laboratory settings. RT-PCR is cost-effective and easy to use for genotyping, and the MassARRAY platform offers scalability with its high multiplex capacity and less stringent DNA quality requirements [6, 22] (Supplemental Table 3). Between November 2021 and August 2022, a global survey on SMA NBS revealed that only four of 21 (19%) surveyed countries had nationwide programs [23]. To reduce the global inequality in managing patients with SMA, countries can choose either RT-PCR or the Sequenom MassARRAY platform for NBS implementation.

SMA1 was the most common phenotype among SMA cohorts in Taiwan, accounting for 52.2% (12/23), followed by SMA3 (13.0%; 3/23) and SMA2 (8.7%; 2/23). Compared to the previous cohort [24], the proportion of type 1 was the same as that in the previous study; however, the ratio of patients with SMA1 with two *SMN2* copies (8/12, 66.7%) was lower than that of the Spanish (235/272, 86.4%) and worldwide (919/1265, 72.6%) series [10].

For the genotype–phenotype correlation, the age at symptom onset in all patients with SMA with two *SMN2* copies was within 62 days (one SMA1a and seven SMA1b). All four patients with SMA1c in our cohort had three *SMN2* copies and exhibited symptoms within 165 days. Our SMA1 cohort reaffirmed that symptoms occurred earlier in patients with SMA1 with two *SMN2* copies than in those with three copies, which is consistent with the natural history of SMA1 [25, 26].

The most common phenotype of infants with three *SMN2* copies in our cohort was SMA1c (36.4%), followed by SMA 3a (27.3%), SMA 2a (18.2%). All patients with three *SMN2* copies became symptomatic within 1 year, except for two children who received presymptomatic DMT. All four patients with four copies were asymptomatic walkers. Our NBS cohort revealed that neonates with SMA1 with two or three *SMN2* copies developed symptoms within 62 or 165 days, respectively, underscoring the need for critical early intervention. In addition, NBS-identified neonates with three *SMN2* copies who later developed SMA2 or SMA3a exhibited symptoms within 1 year of age, also demonstrating the need for timely intervention. These findings highlight the importance of proactive follow-up and prompt treatment strategies for screening-positive neonates with two or three *SMN2* copies at risk of SMA.

In the present study, two patients with SMA1b who received palliative care died at a median age of 8.8 months, consistent with the natural history of SMA1 in Taiwan, where the median disease onset and death are 0.8 and 8.8 months, respectively [26]. Two additional patients with SMA1b who received nusinersen treatment died at a median age of 12.8 months due to acute respiratory failure. This aligns with the findings from the ENDEAR trial, in which 16% (13/80) of patients with SMA1b and SMA1c in the nusinersen group died primarily due to respiratory failure [27]. Among the seven patients with SMA1b and SMA1c treated with DMT, four (57%, 4/7) achieved the ability to walk independently, one (14%, 1/7) could walk with support, and two (29%, 2/7) attained independent sitting. These outcomes have not been observed in the natural history of patients with SMA1 [25, 26]. These findings suggest that NBS combined with DMT can alter the natural history of SMA and significantly improve patient outcomes. However, infants with SMA1a continue to have a poor prognosis despite DMT administration, raising concerns about the timing of treatment initiation and the effectiveness of current SMN replacement therapies in addressing systemic complications [28]. Based on these results, we propose that DMT alone is inadequate for managing patients with SMA1. Proactive multidisciplinary care remains the cornerstone of SMA treatment [29].

Among the two patients with SMA2a and three with SMA3a, four achieved unaided walking and one had the ability to sit without support. Furthermore, two infants at risk for SMA, each with three *SMN2* copies, who received presymptomatic treatment before 20 weeks of age and were followed up for at least two years, achieved asymptomatic walking. Our findings indicate that the early initiation of DMT leads to improved motor function. Outcomes appear to depend on the patient’s initial neurological status and number of *SMN2* copies, which is consistent with observations from other SMA NBS cohorts [5, 30].

Based on the findings of this study, the incidence of SMA in Taiwan was 1 in 19,433 live births, which is consistent with the results of a previous study [6]. This observed incidence was significantly lower than the estimated incidence of SMA based on the carrier rate in Taiwan (1/48) [7]. Over five consecutive years of SMA NBS, we observed a notable decline in the incidence of SMA. (Table 3) This decline may be attributed to the implementation of a nationwide SMA carrier genetic screening program for pregnant women (initiated in January 2005) and the heightened public awareness of SMA following the introduction of DMT in 2020. These findings can guide healthcare providers and genetic counselors to foster patient autonomy and promote shared decision-making in the evolving era of SMA therapeutics [31].

Furthermore, among the DMT-treated patients with two and three *SMN2* copies in our cohort identified through NBS, 62.5% (10/16) could walk independently after treatment, which is comparable to the results in the European cohort (63.6%) [32]. In our cohort, 37.5% of the patients achieved independent walking before 18 months, which is slightly lower than the 40% reported by the SMARTCARE study group [32]. The possible reason is that our cohort had a lower proportion of presymptomatic DMT (12.5% vs. 75%) and the mean age at DMT initiation was later (12.1 months vs. 1.3 months).

According to a previous systematic review of the treatment of patients with SMA identified via NBS, pre-symptomatic intervention has become the predominant approach in real-world clinical settings. The proportions of presymptomatic treatment were 49% and 98% in patients with SMA with two and three *SMN2* copies, respectively [5]. Although DMTs for SMA are costly and not covered by insurance in some developing countries, physicians can explore compassionate-use programs or participate in clinical trials to secure treatment opportunities for patients with SMA, thereby improving outcomes [33–37]. Recent real-world studies, including data from non-randomized controlled trials in Australia and the SMARTCARE registry comprising Germany, Austria, and Switzerland, demonstrated that SMA NBS significantly reduces diagnostic and treatment delays, leading to better outcomes than symptom-based diagnosis and treatment [32, 38].

Adequate government funding or comprehensive insurance coverage for DMT is important to ensure the affordability and long-term sustainability of screening programs [39]. Such financial support not only guarantees equitable access to critical treatments but also helps reduce the barriers faced by affected families. The cost-effectiveness of the SMA NBS program has in many developing countries has been previously reported [39, 40]. Early detection and timely treatment significantly reduce lifelong healthcare expenditures and societal costs compared with the financial burden of untreated cases. The benefits of early intervention are equally compelling. Initiating therapy during the presymptomatic phase, particularly in patients with two or three *SMN2* copies, leads to improved survival rates, enhanced quality of life, and a lower risk of costly complications. These outcomes highlight the importance of SMA NBS as both an economic necessity and a clinical imperative [41]. Our current findings provide real-world epidemiological insights into SMA NBS, the incidence of SMA in the Taiwanese newborn population, and five-year improved outcomes of the SMA NBS program in Taiwan. These findings provide additional supporting evidence for the health economics analysis of SMA NBS programs in other populations. We recommend that countries without nationwide SMA NBS programs consider implementing technologies such as RT-PCR and the MassARRAY platform to expedite NBS and enable the timely initiation of DMT, ultimately improving patient outcomes.

## Conclusions

This study highlighted the long-term outcomes of patients with SMA following the implementation of a five-year nationwide NBS program in Taiwan. Our results confirmed that SMA NBS facilitates early diagnosis and treatment, yielding significantly better outcomes with early DMT administration. Infants identified as at risk for SMA through NBS and received presymptomatic treatment showed superior outcomes compared with those who received symptomatic DMT after diagnosis.

## Supplementary Information

Additional file 1

## Data Availability

All data generated or analyzed during this study are included in this published article and its supplementary information files.

## Abbreviations

SMA: Spinal muscular atrophy
NBS: Newborn screening
RT-PCR: Real-time polymerase chain reaction
MLPA: Multiplex ligation-dependent probe amplification
DMT: Disease-modifying therapy
*SMN1*: Survival motor neuron 1
NHI: National Health Insurance
KMUH: Kaohsiung Medical University Hospital
CFOH: Chinese Foundation of Health
TIP: Taipei Institute of Pathology
DBS: Dried blood spot
CI: Confidence interval
DOL: Days of life
