# Supplementary material for: Newborn screening facilitates early theranostics and improved spinal muscular atrophy outcome: five-year real-world evidence from Taiwan
Source: Orphanet J Rare Dis. 2025 Apr 24;20:197. doi: 10.1186/s13023-025-03697-1 (PMC12023543; doi:10.1186/s13023-025-03697-1)
Supplement: Supplementary file 1 — Additional file 1 [file 13023_2025_3697_MOESM1_ESM.docx]

Supplemental information

**SMA Newborn Screening of the first year**

During the first year of screening (1 September 2017 - 31 August 2018), 110,705 infants were screened for SMA (51.2% by RT-PCR and 48.8% by MassARRAY). In this period, a RT-PCR assay for c.888+100A (Supplemental Figure 1) and a MassARRAY genotyping assay for the 4 *SMN* variants (Supplemental Figure 2) were used. Twenty-two infants were screened positive (Supplemental Table 1; 10 by RT-PCR and 12 by MassARRAY). For the 12 infants identified by MassARRAY, 7 had a homozygous deletion of *SMN1* exon 7 to exon 8, whereas the remaining 5 retained *SMN1* exon 7 but lost parts of *SMN1* (1 lost intron 7 and exon 8, 4 lost exon 8). The 5 infants with loss of partial *SMN1* were referred for further testing and clinical evaluation.

**Cross validation of screened positive samples**

The 22 screened positive DBSs were exchanged to be tested using different screening methods (Supplemental Table 1). A total of 5 DBSs had inconsistent results (Supplemental Table 1). One DBS RT-PCR positive had a negative result for the 4 *SMN* variants determined by MassARRAY. The other 4 MassARRAY screened positive DBSs had negative results by c.888+100A RT-PCR.

**MLPA confirmatory diagnosis**

The MLPA results confirmed that 13 of the 22 infants lacked *SMN1* (Supplemental Table 1) and were diagnosed with presymptomatic SMA, while the remaining 9 infants contained one copy of *SMN1* and were carriers (4 found by RT-PCR and 5 detected by MassARRAY). To exclude point mutations on *SMN1* gene, *SMN1* gene of the 9 infants were sequenced. Other than c.-39A>G identified in 4 infants (Supplemental Table 1); no sequence variants were identified. Molecular test results revealed that the 9 infants were screened false-positive (FP).

**Screened false-positive infants**

The 9 FP DBSs (5 and 4 exhibited inconsistent and consistent screening findings, respectively) were subjected to *SMN* PCR and sequenced using *SMN1* specific primer to ascertain the cause of false positive. Three conditions were identified, including a sequence alteration and presence of *SMN1*-*SMN2* hybrid alleles. One infant had a c.888+102A>C alteration in the TaqMan binding site, which resulted in a false positive. Two forms of *SMN1*-*SMN2* hybrid alleles produced by gene conversions were identified in 8 infants (Supplemental Figure 3).

The c.-39A>G alteration was identified in the 4 infants with type 1 hybrid (Supplemental Table 1), which was absent in the 13 infants lacking *SMN1* (Fisher’s exact test, p<0.01). The carrier rate of c.-39A>G in the general population was 1.3% (95% CI, 0.62%-2.62%, data not shown), indicating that c.-39A>G is a polymorphism co-segregating with type 1 hybrid alleles rather than a disease-causing mutation. All the 9 cases were clinically follow-up and remained asymptomatic.

In a summary of the first year's screening results, false positives in the RT-PCR and MassARRAY assays were caused by technique and interpretation criteria, respectively. Identification of false positive by RT-PCR was eliminated by changing the design of primers and probe. The specificity of RT-PCR increased from 99.99% (95% CI, 99.98%-100.00%) to 100.00% (95% CI, 100.00%-100.00%) after changing the primers and probe, the false positive rate (FPR) decreased from 0.007% to 0.000%, and the positive predictive value increased from 60.0% to 100.0%. Performance for SMA NBS using RT-PCR improved (Supplemental Table 2).

False positive infants with hybrid alleles identified by MassARRAY were due to more stringent positive interpretation criteria. Interpretation using c.840 genotype eliminated unnecessary referrals for carriers carrying *SMN1*-*SMN2* hybrids. However, 50% FP (6/12) was observed in the later study. The 6 infants had a c.835-24dup variation, which might interfere with the detection of c.840C. After altering the interpretation criteria, MassARRAY's specificity stayed at 99.99% (95% CI, 99.99%-100.00%), the false positive rate (FPR) dropped from 0.009% to 0.002%, and the positive predictive value dropped from 58.3% to 50.0%. Overall, the existing approaches appear to have an exceedingly low false positive rate.

**Supplemental Figure 1. SMA newborn screening using TaqMan Real-Time PCR (RT-PCR) assays**

(A) Diagram of the c.888+100A RT-PCR detection marker on *SMN1* intron 7. (B) The results of a standard RT-PCR genotyping assay. The green curves represented samples with undetectable amplification signal, such as SMA patients with zero copy *SMN1* and negative template control (NTC). The red curves represented samples that had one or more copies of *SMN1*. Samples with and without the *SMN1* gene could be easily differentiated. (C) Validation of c.888+100A RT-PCR using DBSs from anonymous infants (N = 3,079), SMA carriers (N = 3), SMA patients with homozygous deletion (N = 7) and a patient with point mutation. The Cp values for samples containing the *SMN1* gene (copy number ≥1) were ranged between 20 and 30, whereas Cp values for samples without the *SMN1* were undetectable.


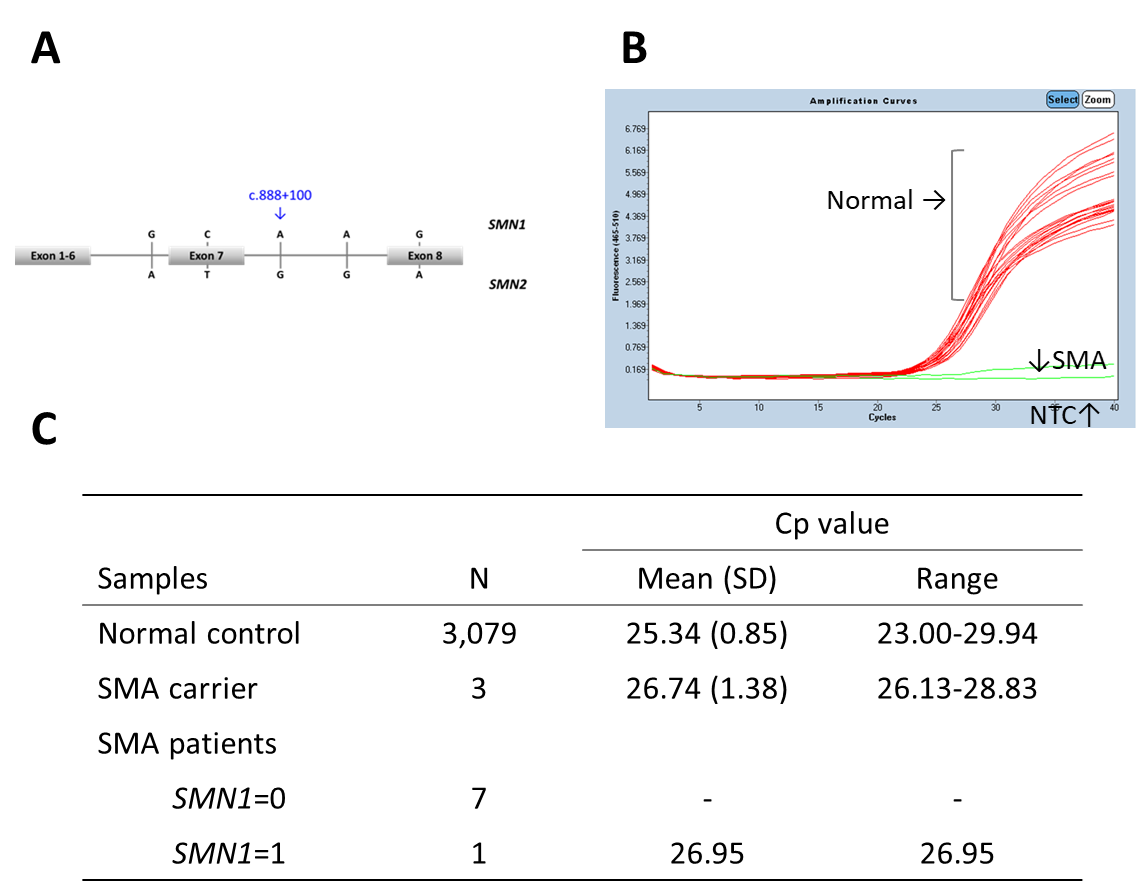


**Supplemental Figure 2. SMA newborn screening using MassARRAY assay**

(A) Schematic of position of the 4 *SMN1*/*2* variations detected. (B) A normal control's exon 7 mass spectra. Both peaks representing *SMN1* (c.840C) and *SMN2* (c.840T) were observed. (C) The mass spectra of exon 7 from a SMA patient. Only the *SMN2* (c.840T) peak was identified. The red and blue lines on the right side indicated the nucleotide in heterozygous and homozygous status, respectively. The red line on the left side indicated mass of unextended primers. (D) The *SMN1/2* MassARRAY test was validated on anonymous DBSs from infants (N = 2,125) and SMA patients lacking *SMN1* (N = 3). To determine the presence of the *SMN1* allele, the relative ratio of the *SMN2* allele's area to the sum of the *SMN1* and *SMN2* allele's areas was used. A clear discrimination between samples with and without *SMN1* could be made. For normal and SMA patients, the *SMN* exon 7 ratios were <0.85 and nearly 1, respectively.


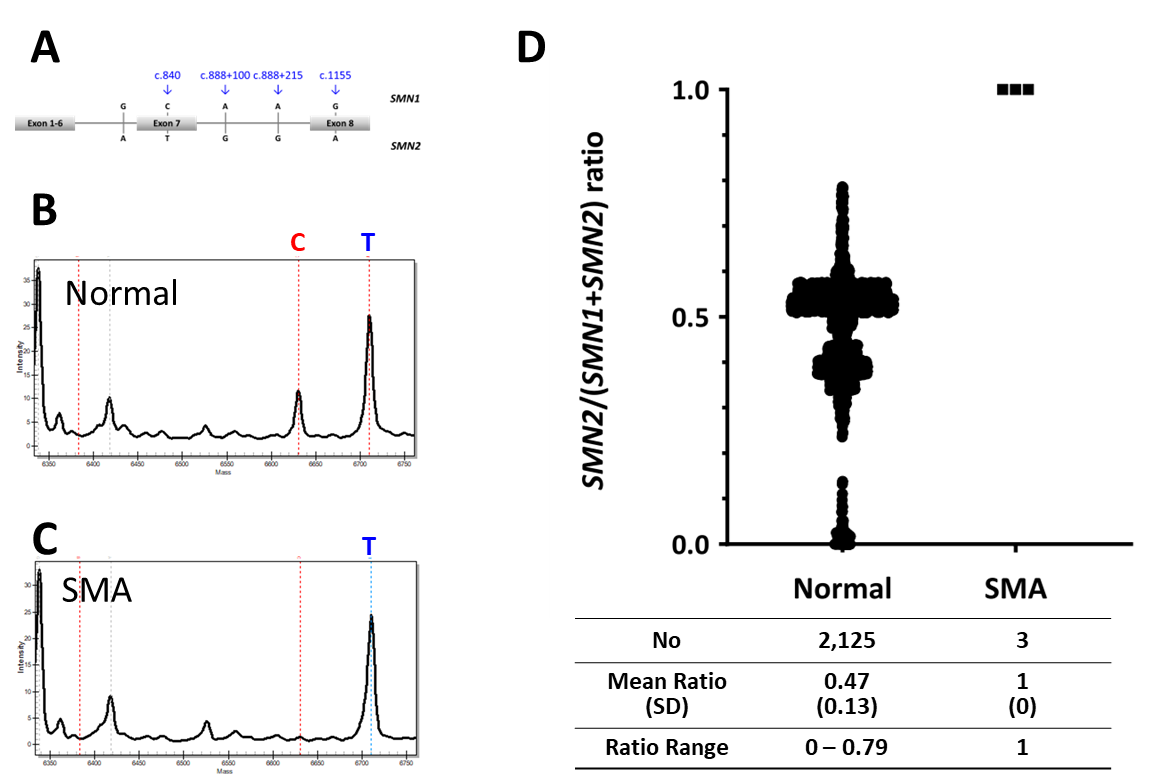


**Supplemental Figure 3 Schematic illustration of the *SMN1*-*SMN2* hybrid alleles**

Two types of *SMN1*-*SMN2* hybrids resulted from different gene conversions were identified. The intron 7 of *SMN2* was fused to exon 7 of *SMN1*, yielding a type 1 hybrid with the genotypes c.840C (*SMN1*), c.888+100G (*SMN2*), c.888+215G (*SMN2*), and c.1155A (*SMN2*). The fusion of exon 8 of *SMN2* into intron 7 of *SMN1* produced a type 2 hybrid with the genotypes c.840C (*SMN1*), c.888+100A (*SMN1*), c.888+215A (*SMN1*), and c.1155A (*SMN2*).


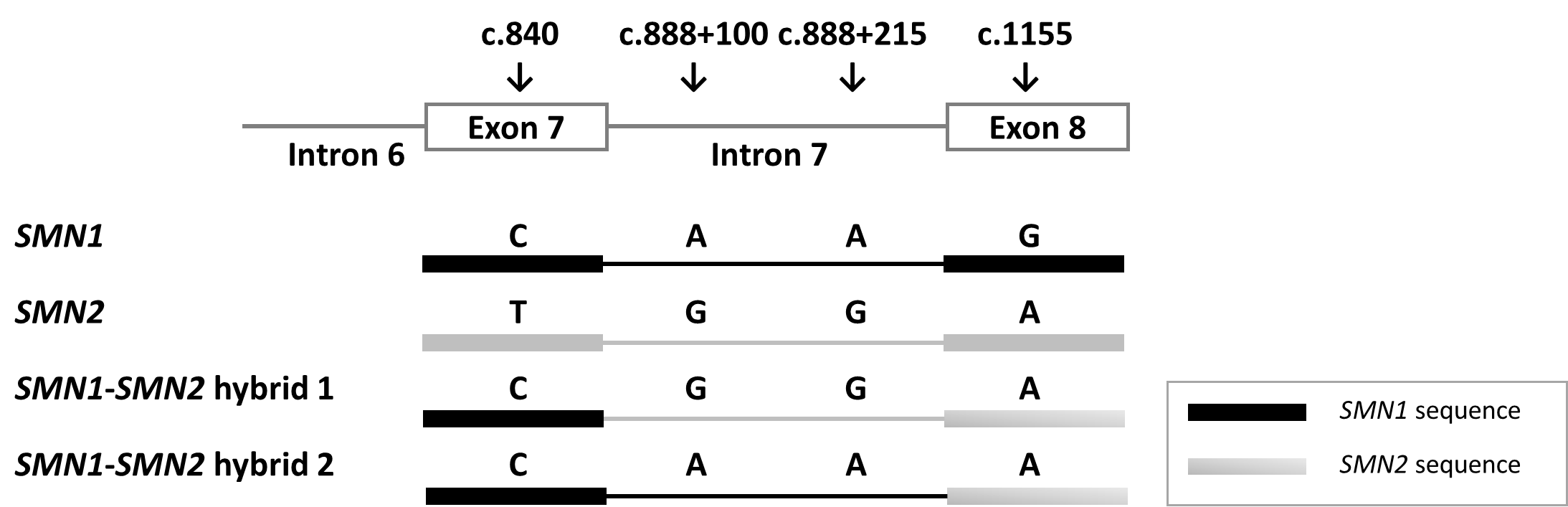


**Supplemental Table 1. Summary of the 22 NBS-positive samples' cross-validation, Sanger sequencing, and MLPA confirmatory results**

| NBS |  | *SMN* Variants Genotype^2^ | | | |  | Screening Results^3^ | | | | | Exon 7 Copy No^4^ | Other Variants |
| --- | --- | --- | --- | --- | --- | --- | --- | --- | --- | --- | --- | --- | --- |
| Center | Cases | c.840 | c.888+100 | c.888+215 | c.1155 |  | CFOH |  | TIP |  | Consistency | *SMN1*: *SMN2* | Identified |
| CFOH | 1 | T | G | G | A |  | + |  | + |  | Y | 0:3 |  |
|  | 2 | T | G | G | A |  | + |  | + |  | Y | 0:3 |  |
|  | 3 | T | G | G | A |  | + |  | + |  | Y | 0:2 |  |
|  | 4 | T | G | G | A |  | + |  | + |  | Y | 0:3 |  |
|  | 5 | T | G | G | A |  | + |  | + |  | Y | 0:2 |  |
|  | 6 | T | G | G | A |  | + |  | + |  | Y | 0:2 |  |
|  | FP1^1^ | C/T | A/G | A/G | G/A |  | + |  | - |  | N | 1:3 | c.888+102A>C |
|  | FP2^1^ | C/T | G | G | A |  | + |  | + |  | Y | 1:3 | c.-39A>G |
|  | FP3^1^ | C/T | G | G | A |  | + |  | + |  | Y | 1:3 | c.-39A>G |
|  | FP4^1^ | C/T | G | G | A |  | + |  | + |  | Y | 1:1 | c.-39A>G |
| TIP | 10 | T | G | G | A |  | + |  | + |  | Y | 0:3 |  |
|  | 11 | T | G | G | A |  | + |  | + |  | Y | 0:2 |  |
|  | 12 | T | G | G | A |  | + |  | + |  | Y | 0:4 |  |
|  | 13 | T | G | G | A |  | + |  | + |  | Y | 0:2 |  |
|  | 14 | T | G | G | A |  | + |  | + |  | Y | 0:3 |  |
|  | 15 | T | G | G | A |  | + |  | + |  | Y | 0:3 |  |
|  | 16 | T | G | G | A |  | + |  | + |  | Y | 0:4 |  |
|  | FP5^1^ | C/T | G | G | A |  | + |  | + |  | Y | 1:2 | c.-39A>G |
|  | FP6^1^ | C/T | A/G | A/G | A |  | - |  | + |  | N | 1:3 |  |
|  | FP7^1^ | C/T | A/G | A/G | A |  | - |  | + |  | N | 1:2 |  |
|  | FP8^1^ | C/T | A/G | A/G | A |  | - |  | + |  | N | 1:3 |  |
|  | FP9^1^ | C/T | A/G | A/G | A |  | - |  | + |  | N | 1:2 |  |

1. Screened false positive cases.
2. Genotypes were confirmed using Sanger sequencing. Haplotype for *SMN1*: c.840C, c.888+100A, c.888+215A, c.1155G; haplotype for *SMN2*: c.840T, c.888+100G, c.888+215G, c.1155A.
3. c.888+100A RT-PCR and MassARRAY assays were used by CFOH and TIP, respectively. +, screened positive; -, screened negative; Y: yes; N: no
4. Copy numbers of the *SMN1* and *SMN2* were confirmed by MLPA assay.

**Supplemental Table 2. Screening procedures modifications improved SMA screening performance**

| NBS center | CFOH | |  | TIP | |
| --- | --- | --- | --- | --- | --- |
| Period | 1^st^ year | 2^nd^-5^th^ year |  | 1^st^ year | 2^nd^-5^th^ year |
| Confirmed SMA | 6 | 3 |  | 7 | 6 |
| False Positive Case | 4 | 0 |  | 5 | 6 |
| False Negative Case | 0 | 0 |  | 0 | 0 |
| Total NB Screened | 56,730 | 179,483 |  | 53,975 | 156,777 |
| Sensitivity  (95% CI) | 100.00%  (60.97%-100.00%) | 100.00%  (43.85%-100.00%) |  | 100.00%  (64.57%-100.00%) | 100.00%  (60.97%-100.00%) |
| Specificity  (95% CI) | 99.99%  (99.98%-100.00%) | 100.00%  (100.00%-100.00%) |  | 99.99%  (99.98%-100.00%) | 99.99%  (99.99%-100.00%) |
| False positive rate | 0.007% | 0.000% |  | 0.009% | 0.002% |
| Positive predictive value | 60.0% | 100.0% |  | 58.3% | 50.0% |

**Supplemental Table 3 Methods used for SMA newborn screening**

| NBS centers | CFOH | TIP |
| --- | --- | --- |
| Method | Real time-PCR | MassARRAY |
| Marker | c.888+100A/  c.840C | c.840  c.888+100  c.888+215  c.1155 |
| Turn-around time (day) | 7 | 7 |
| Maximum throughput (tests per run) | 384 | 384 |
| Reagent cost per test (USD) | ≤3 | 6 |
| Advantage | 1. Cost-effectiveness for genotype determination 2. Easy to operate and interpret data | 1. Scalability due to high multiplex capacity 2. DNA quality requirements are not stringent |
